# Effects of fear of missing out on inhibitory control in social media context: evidence from event-related potentials

**DOI:** 10.3389/fpsyt.2023.1301198

**Published:** 2023-11-10

**Authors:** Yang Xu, Yu Tian

**Affiliations:** ^1^Institute of Brain and Psychological Sciences, Sichuan Normal University, Chengdu, China; ^2^Sichuan Key Laboratory of Psychology and Behavior of Discipline Inspection and Supervision (Sichuan Normal University), Chengdu, China

**Keywords:** fear of missing out, inhibitory control, event-related potentials, social media, two-choice oddball task

## Abstract

The present study aimed to investigate the impact of fear of missing out (FoMO) on inhibitory control in social media context. The present study used a two-choice oddball task combined with event-related potentials (ERPs) technology to measure inhibitory control. Based on the Fear of Missing Out Scale, participants with varying degrees of FoMO were recruited to complete two studies. A total of 78 participants in Study 1 completed a two-choice oddball task (stimuli “W” or “M”). The results showed that FoMO did not have a significant impact on general inhibitory control at both the behavioral and electrophysiological levels. To further examine the effect of FoMO in social media context. In Study 2, 72 participants completed a modified two-choice oddball task with three types of pictures (high and low social media-related and neutral). The behavioral results revealed that as FoMO scores increased, inhibitory control decreased. ERP analysis revealed that with higher FoMO scores, social media-related pictures elicited larger N2 amplitude and smaller P3 amplitude, but not for neutral pictures. This suggests that FoMO undermines inhibitory control by consuming more cognitive resources in the early conflict detection stage and leading to insufficient cognitive resources in the later stages of the inhibitory process. These findings suggest that FoMO can undermine inhibitory control in the social media context. Considering the indispensable use of social media in the digital age, addressing and understanding the influence of FoMO on inhibitory control could be essential for promoting healthy digital behaviors and cognitive functions.

## 1. Introduction

As social media continues to advance rapidly, it has become the primary platform for interpersonal interaction (1). Recently, researchers have found that using social media may induce fear of missing out (FoMO), as individuals worry about potentially missing rewarding experiences such as interesting things and important notifications (2, 3). Przybylski et al. (3) found that individuals with FoMO tend to be easily distracted by engaging in social media activities, resulting in a lack of attention (e.g., distracted driving and studying). Previous research has demonstrated that distractions can diminish attentional control during ongoing tasks, thereby undermining individuals’ inhibitory control (4, 5). Inhibitory control is a critical cognitive process that involves the ability to restrain from engaging in inappropriate or unnecessary behaviors (6). Issues related to inhibitory control can contribute to problematic social media use and negatively impact one’s quality of life (7, 8). While numerous studies have illustrated the impact of FoMO on psychological aspects and social media abuse (2, 9, 10), whether and how FoMO affects inhibitory control remains unclear.

Previous studies have investigated a wealth of evidence suggesting that negative emotions (e.g., anxiety, disgust, and fear) can affect inhibitory control (11–13). For example, Xia et al. (12) found that individuals with high-trait anxiety exhibited poorer accuracy and longer reaction times compared to those with low-trait anxiety. As inhibitory control involves multiple processes, such as conflict detection and response inhibition (14, 15). Event-related potentials (ERPs) with high temporal resolution have been recommended as a method for investigating inhibitory control. Two related ERP components were employed to assess the processes of inhibitory control. The first component is the N2, a negative waveform emerging approximately 200–400 ms after the signal presentation with a frontocentral distribution. The N2 serves as an indicator of the early stages of the inhibitory process (14). The N2 amplitude was interpreted as the cognitive resources consumed by the earlier conflict detection (16, 17). Another component is the P3, a positive waveform arising from 300 to 600 ms after signal onset with a parietal-central distribution. The P3 reflects the late stage of the inhibitory process response and is associated with the actual inhibition of the motor system (18–20). The increase in P3 amplitude indicates that more cognitive resources need to be consumed to invest in the response process (12).

A previous study found that individuals with high-trait anxiety exhibit smaller N2 amplitude and larger P3 amplitude compared to those with low-trait anxiety (12). Furthermore, Wei et al. (16) found that individuals with high test anxiety exhibited a larger N2 amplitude than those with low test anxiety in the performance evaluation threat conditions. This increase in N2 amplitude was associated with heightened top-down attentional control resources in individuals with high test anxiety. Xu et al. (13) found that the P3 amplitude in disgusting contexts was smaller than in fearful contexts, as disgust consumes more attentional resources during the early stages of the inhibitory process. FoMO also presents such a feature. Individuals experiencing FoMO may worry about missing out on rewarding experiences on social media. When limited attention is diverted to rewarding information or experiences, subsequent inhibitory control might be impaired. Recently, researchers have found that high levels of FoMO in individuals may affect executive function, leading to more impulsivity (21). Impulsivity is highly correlated with inhibitory control, and impulse control problems stem from difficulties with inhibitory control (22).

Previous studies have primarily examined inhibitory control through the Go/NoGo task. In this task, participants are typically instructed to respond quickly and accurately to the Go stimulus while refraining from responding to the NoGo stimuli (23). However, it is important to note that Go trials require motor responses, while NoGo trials do not. Consequently, the observed effects of inhibitory control in studies utilizing the Go/NoGo task are susceptible to the influence of response-related processes (24). This effect may be more pronounced in ERP studies, as P3 components are especially sensitive to movement-related potentials (20). Therefore, the present study chose to use a two-choice oddball task that requires participants to respond accurately and quickly to high-frequency standard stimuli and low-frequency deviant stimuli. Because participants must respond to both types of stimuli, the results remain uncontaminated by motor response-related processes. Research has demonstrated that this task is equally effective in inducing inhibitory control as the Go/NoGo task (25–28).

Overall, the present study aimed to examine how FoMO affects inhibitory control. According to the Interaction of Person-Affect-Cognition-Execution (I-PACE) model, researchers have classified inhibitory control into two types: general inhibitory control and stimulus-specific inhibitory control (6). Compared to general inhibitory control, stimulus-specific inhibitory control is often used to explain inhibitory control deficits that occur when facing addiction-related cues (15, 29). Therefore, two studies were conducted using a two-choice oddball task combined with ERPs to explore the impact of FoMO on inhibitory control. Study 1 used a two-choice oddball task to investigate general inhibitory control. We hypothesized that FoMO would affect general inhibitory control. Furthermore, previous studies have demonstrated that relevant cues can lead to a decline in inhibitory control (23, 30). FoMO is often associated with social media (2). Thus, Study 2 used a modified two-choice oddball task with social media-related cues to assess stimulus-specific inhibitory control in social media context. We hypothesized that FoMO would affect stimulus-specific inhibitory control in the social media context, especially for highly social media-related pictures.

## 2. Study 1

### 2.1. Methods

#### 2.1.1. Participants

A total of 80 participants with varying degrees of FoMO were recruited. Every participant had a history of over 5 years of social media use. Two participants were excluded due to a large number of ocular artifacts. As a result, the data analysis included 78 participants, consisting of 35 male participants (44.87%). There was no significant difference in FoMO scores between genders (*p* > 0.05) (*M*_male_ = 24.91, *M*_female_ = 24.41). The mean age was 20.31 years (SD = 2.03). Participants received comprehensive instructions and provided written informed consent before the study. The study was approved by the local Institutional Ethical Committee.

#### 2.1.2. Measures and procedure

##### 2.1.2.1. Fear of missing out

FoMO was measured by the Fear of Missing Out Scale (3). This 10-item measure asked participants to rate how true each statement was of their general experiences (1 = “not at all true of me” and 5 = “extremely true of me”). The higher score indicates a greater fear of missing out. Cronbach’s *α* was 0.85 in this study.

##### 2.1.2.2. Two-choice oddball task

Before the experiment, participants completed the necessary scales. Then, they underwent a 20-trial familiarization phase to become acquainted with the task. The formal experiment commenced only when participants achieved 100% accuracy in the practice trials.

**Figure 1:**
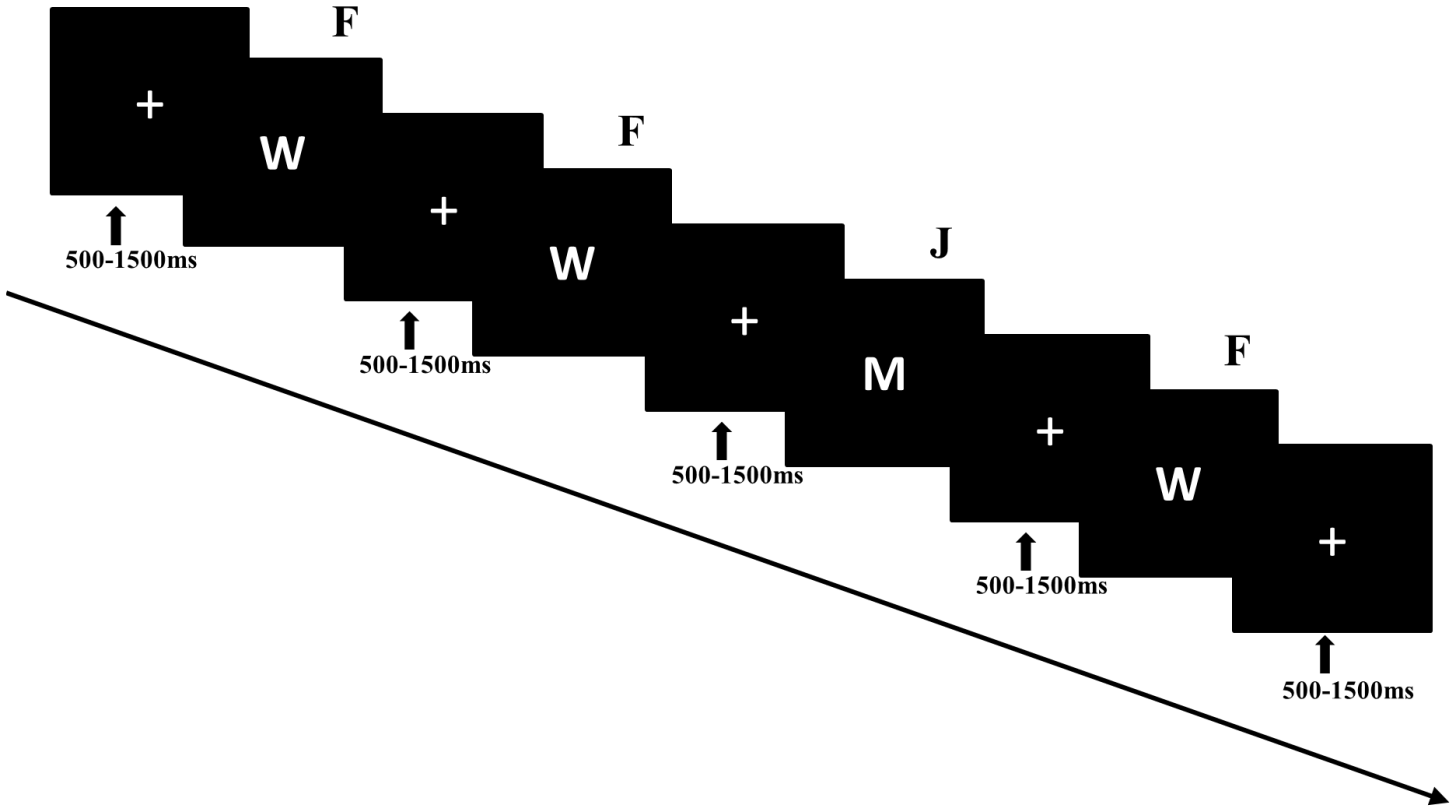
Sequence of events in the experimental trial and an example of standard stimuli and deviant stimuli. Each trial presented a single stimulus.

The experiment consisted of 5 blocks, each comprising 120 trials. Each trial commenced with the presentation of a small white cross, and its duration varied randomly between 500 and 1,500 ms. Next, the stimulus was presented. For half of the participants, if the standard stimulus (“W”; 80% of trials) was presented, they were instructed to press the “F” key with their left index finger as quickly as possible. If the deviant stimulus (“M”; 20% of trials) was presented, they were to press the “J” key with their right index finger. The stimulus vanished upon keypress or when 1,000 ms had passed (see Figure 1). The response keys were reversed for the other half of the participants.

#### 2.1.3. Electrophysiological recording

The EEG was recorded using the ANT-NEURO system (Enschede, The Netherlands) with 64 Ag/AgCl electrodes arranged in a 10/20 system layout (AFz serving as ground and CPz serving as the online reference). An electrooculogram (EOG) was recorded by electrodes placed on the outer canthi of the left eye to detect blinks. The impedances of the electrodes were maintained below 5 kΩ. Data were digitized at 500 Hz.

We used the EEGLAB toolbox within the MATLAB software for EEG data analysis. We applied a band-pass filter ranging from 0.01 Hz to 40 Hz to the data. The reference electrode standardization technique (REST) was adopted as a re-reference method (31). Ocular artifacts and head movement were eliminated using independent component analysis (ICA). The EEG data were segmented into epochs, starting with 200 ms before stimulus onset and continuing until 800 ms (i.e., −200 to 800 ms). The period of 200 ms pre-stimulus was used as the baseline to align ERP amplitude. Any trials displaying ERP sweeps with amplitudes exceeding ±80 μV were excluded from the analysis.

**Figure 2:**
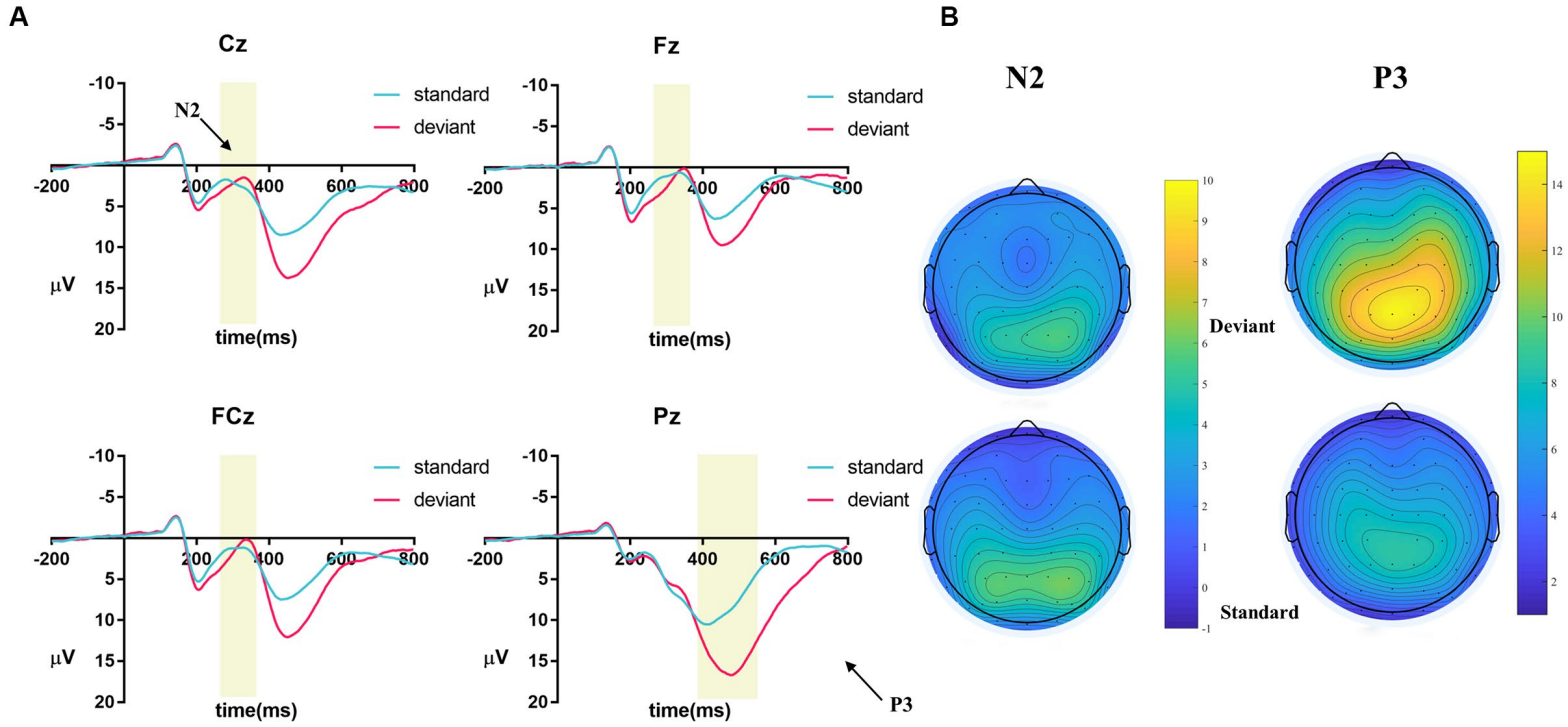
**(A)** ERPs in different picture types (deviant and standard) at electrode points (Fz, Cz, FCz, and Pz); **(B)** topographic maps for N2 (260–360 ms) and P3 (380–550 ms).

To separate the inhibitory control components, the ERPs under the two stimuli were subtracted (deviant − standard) to obtain the difference waves between the two stimulus conditions. Based on the previous studies (12, 16, 23) and the observation of the grand-averaged ERP waveforms and topographic maps (see Figures 2A,B), we analyzed the mean amplitudes of the frontal N2 and parietal P3. The N2 was measured using the mean amplitude at the electrode points of the Fz, Cz, and FCz within a time window of 260–360 ms. P3 was measured using the mean amplitude at the Pz electrode point within a time window of 380–550 ms.

#### 2.1.4. Statistics analysis

##### 2.1.4.1. Behavioral analysis

A repeated-measures ANOVA with stimulus type as a within-subject factor was performed for accuracy (ACC) and reaction times (RTs) respectively. The ACC cost was calculated as the difference in ACC between standard and deviant trials (standard – deviant). The RT cost was calculated as the difference in reaction time between deviant and standard trials (deviant – standard). Higher values of both ACC cost and RT cost may indicate a decline in inhibitory control.

##### 2.1.4.2. ERP analysis

A repeated-measures ANOVA was used to compare the mean amplitudes of the N2 and P3 components. For N2, the two within-subject variables were trial type (standard and deviant) and the three electrode points (Cz, Fz, and FCz). For P3, the two within-subject variables were trial type (standard and deviant) and the electrode point (Pz).

All data analyses were performed using SPSS 25.0; Bonferroni correction was used to correct for multiple comparisons in *post-hoc* tests. All statistical values were reported with Greenhouse–Geisser corrections.

## 3. Results

### 3.1. Behavior results

#### 3.1.1. ACC

The repeated-measures ANOVA revealed a significant main effect for trial type [*F* (1, 77) = 78.85, *p* < 0.001, *η*_p_^2^ = 0.51]. The ACC of the deviant stimuli (95.19%) was significantly lower than that of the standard stimuli (99.27%).

#### 3.1.2. RT

**Figure 3:**
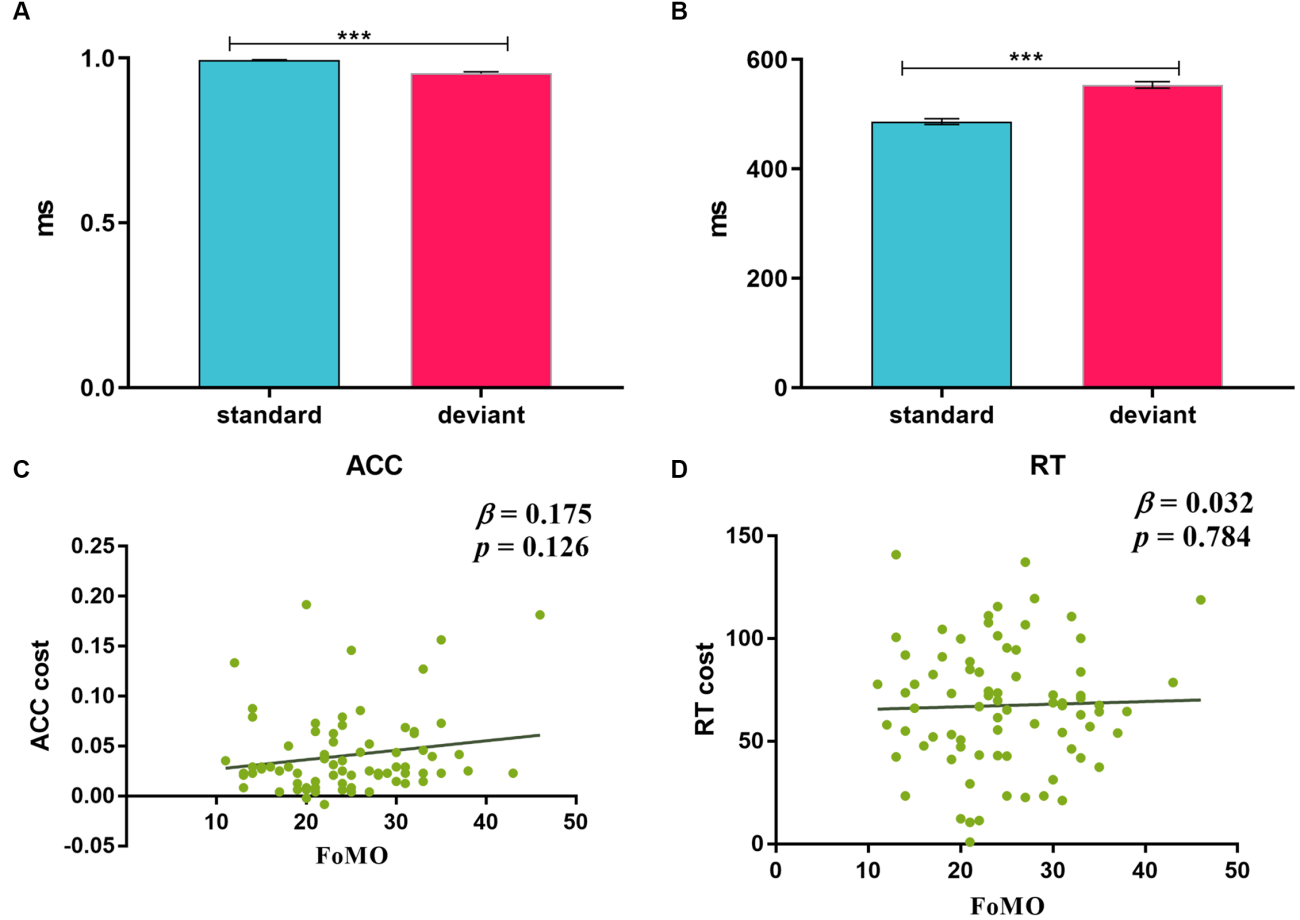
**(A,B)** ACC and RT for standard and deviant stimuli; **(C,D)** regression analyses between FoMO and ACC cost and RT cost. Bars represent the standard error of the mean, the same as below.

The repeated-measures ANOVA of RT on correct trials showed a significant main effect for trial type [*F* (1, 77) = 390.76.1, *p* < 0.001, *η*_p_^2^ = 0.84], which indicated faster RT for the standard stimuli (486.35 ms) than that for the deviant stimuli (553.77 ms) (see Figures 3A,B).

Linear regression was used to examine whether FoMO scores could predict ACC cost and RT cost. The results showed that FoMO scores did not significantly predict ACC cost and RT cost (*p*-values > 0.05) (see Figures 3C,D).

### 3.2. ERP results

N2 and P3 amplitudes and topographic maps are displayed in Figures 2A,B.

#### 3.2.1. N2 component

The repeated-measures ANOVA revealed that the main effect of trial type was not significant (*p* > 0.05). The main effect of electrode points was significant [*F* (1, 77) = 12.99, *p* < 0.001, *η*_p_^2^ = 0.14], and the trial type × electrode points was significant [*F* (2, 154) = 59.13, *p* < 0.001, *η*_p_^2^ = 0.43]. The linear regression was used to examine whether FoMO scores could predict the N2 amplitude (deviant – standard). The results showed that FoMO scores did not significantly predict N2 amplitude (Cz, Fz, and FCz) (*p*-values > 0.05).

#### 3.2.2. P3 component

The repeated-measures ANOVA revealed that the main effect of trial type was significant [*F* (1, 77) = 239.32, *p* < 0.001, *η*_p_^2^ = 0.76], suggesting that participants revealed a significantly larger P3 amplitude for the deviant stimuli (*M* = 14.67 μV) than the standard stimuli (*M* = 8.47 μV). The linear regression was used to examine whether FoMO scores could predict the P3 amplitude (deviant – standard). The results showed that FoMO scores did not significantly predict P3 amplitude (Pz) (*p* > 0.05).

## 4. Discussion

Based on the current study, no effects of FoMO on general inhibitory control have been observed at both behavioral and electrophysiological levels. The N2 reflects the conflict monitoring of irrelevant information (32). In Study 1, the difference in N2 amplitude between the two stimulus types was not significant. Simplified stimuli may not evoke their concerns about missing out on rewarding information or experiences. Previous research has demonstrated that relevant cues can lead to a decline in stimulus-specific inhibitory control (23, 33). FoMO is often associated with social media use and constant exposure to others’ activities and achievements (10). Study 2 added social media-related cues to further examine the effect of FoMO on stimulus-specific inhibitory control.

## 5. Study 2

### 5.1. Methods

#### 5.1.1. Participants

Seventy-eight participants were included in the data analysis, among whom six were excluded due to a high error rate and a large number of ocular artifacts. As a result, 72 participants were included in the data analysis, which comprised 34 male participants (47.22%). The mean age was 20.42 years (SD = 2.08).

#### 5.1.2. Materials

The stimuli consisted of two types: a natural scene of a lamp served as the standard stimulus (70%). The deviant stimuli comprised three kinds of pictures: high social media (HSM; 10%), low social media (LSM; 10%), and neutral pictures (10%).

Each category contained 40 pictures (260 × 260 pixels). The neutral pictures were sourced from the International Affective Picture System (IAPS). The HSM stimuli comprised icons related to HSM (e.g., WeChat and TikTok). The LSM stimuli comprised icons related to LSM (e.g., Map and Music Player). Before the formal experiment, 36 additional participants (16 male participants, *M*_age_ = 21.97 ± 2.71 years) were recruited to rate each picture on valance (1 = very unpleasant; 5 = no apparent pleasant or unpleasant experience; and 9 = very pleasant), arousal (1 = very relaxing and 9 = very exciting), familiarity (1 = very familiar and 9 = very unfamiliar), and social media relevance (1 = not at all relevance and 9 = very relevance) on a 9-point scale. Based on these ratings, 30 pictures were selected for each category in total. The scores for the picture types are presented in Table 1.

**Table 1:** Scores of stimuli pictures (*M* ± SD).

Rating items	Neutral	HSM	LSM
Valence	5.45 ± 0.63	5.44 ± 0.79	5.39 ± 0.46
Arousal	4.11 ± 0.81	4.54 ± 1.03	4.10 ± 0.80
Familiarity	5.43 ± 1.16	5.60 ± 1.85	4.87 ± 1.57
Social media relevance	2.63 ± 0.68	6.28 ± 1.08	5.26 ± 0.70

The repeated-measures ANOVA revealed that the main effects of valence, arousal, and familiarity were not significant (*p*-values > 0.05). The main effect of social media relevance was significant [*F* (2, 58) = 166.01, *p* < 0.001, *η*_p_^2^ = 0.85]. *Post-hoc* analyses revealed that social media relevance scores were higher for the HSM pictures than for the LSM and neutral pictures.

#### 5.1.3. Measures and procedure

Participants performed 20 trials prior to the experiment. The formal experiment began only when performance on the practice trials was 100% accurate.

**Figure 4:**
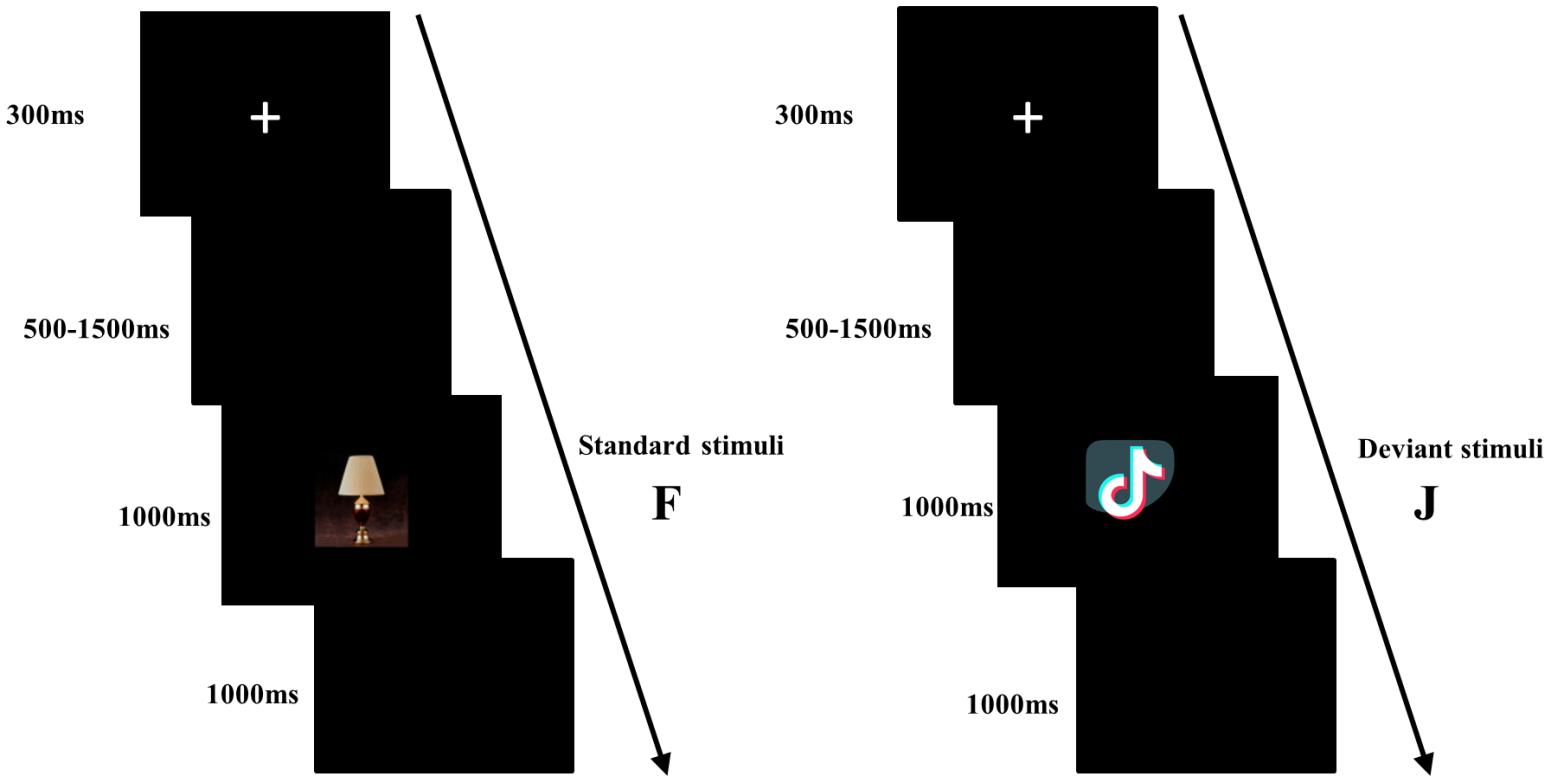
Sequence of events in the experimental trial and an example of standard stimuli and deviant stimuli.

In the present study, a modified oddball task was employed. The experiment consisted of 5 blocks, each comprising 120 trials. Each trial began with a small white cross displayed for 300 ms. A blank screen was then presented for a randomly varying duration between 500 and 1,500 ms. The picture stimulus then appeared. When the standard picture appeared, the participants needed to quickly and accurately press the “F” on the keyboard with their left index finger, and when the deviation picture appeared, they needed to press the “J” key with their right index finger (keyboard keys were balanced between participants). The stimulus picture disappeared after the key press or if 1,000 ms elapsed. Each response was followed by 1,000 ms of a blank screen (see Figure 4).

#### 5.1.4. Statistics analysis

##### 5.1.4.1. Behavioral analysis

A repeated-measures ANOVA with stimulus type as a within-subject factor was performed for ACC and RT. For deviant stimuli, picture type was analyzed as a within-subject factor for ACC and RT.

##### 5.1.4.2. ERP analysis

A repeated-measures ANOVA was used to compare the mean difference amplitudes of the N2 and P3 components. For N2, the two within-subject variables were picture type (HSM, LSM, neutral) and the three electrode points (Cz, Fz, and FCz). For P3, the two within-subject variables were picture type (HSM, LSM, and neutral) and the electrode point (Pz).

All data analyses were performed using SPSS 25.0; Bonferroni correction was used to correct for multiple comparisons in *post-hoc* tests. All statistical values were reported with Greenhouse–Geisser corrections.

## 6. Results

### 6.1. Behavior results

#### 6.1.1. ACC

The repeated-measures ANOVA revealed a significant main effect for trial type [*F* (1, 71) = 22.19, *p* < 0.001, *η*_p_^2^ = 0.24]. The ACC of the deviant stimuli (97.13%) was significantly lower than that of the standard stimuli (98.41%). In the deviant stimuli, there was a significant main effect for picture type [*F* (2, 142) = 12.57, *p* < 0.001, *η*_p_^2^ = 0.15]. The ACC in the conditions of HSM (97.64%) and LSM (97.59%) stimuli was higher than those of neutral stimuli (96.16%).

#### 6.1.2. RT

**Figure 5:**
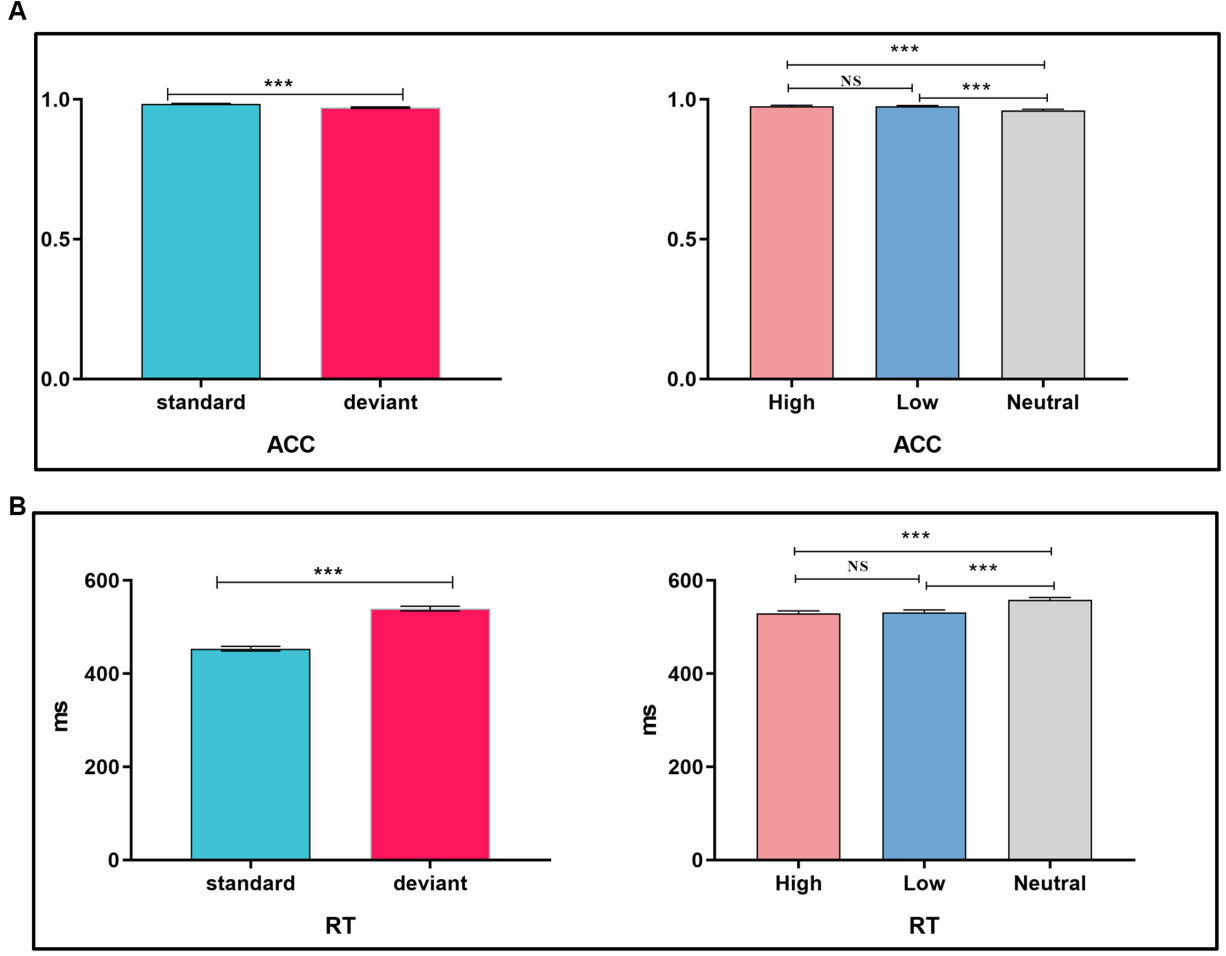
**(A)** ACC for trial types (standard and deviant) and picture types (high, low, and neutral); **(B)** RT for trial types (standard and deviant) and picture types (high, low, and neutral).

The repeated-measures ANOVA of RT on correct trials showed a significant main effect for trial type [*F* (1, 71) = 553.49.1 *p* < 0.001, *η*_p_^2^ = 0.89], with longer RT on correct trials for deviant stimuli (539.78 ms) than on those for standard stimuli (453.93 ms). For deviant stimuli, there was a significant main effect for picture type [*F* (2, 142) = 149.23, *p* < 0.001, *η*_p_^2^ = 0.68]. The RTs in the condition of HSM (529.55 ms) and LSM (531.55 ms) stimuli were lower than that of neutral stimuli (558.25 ms) (see Figures 5A,B).

**Figure 6:**
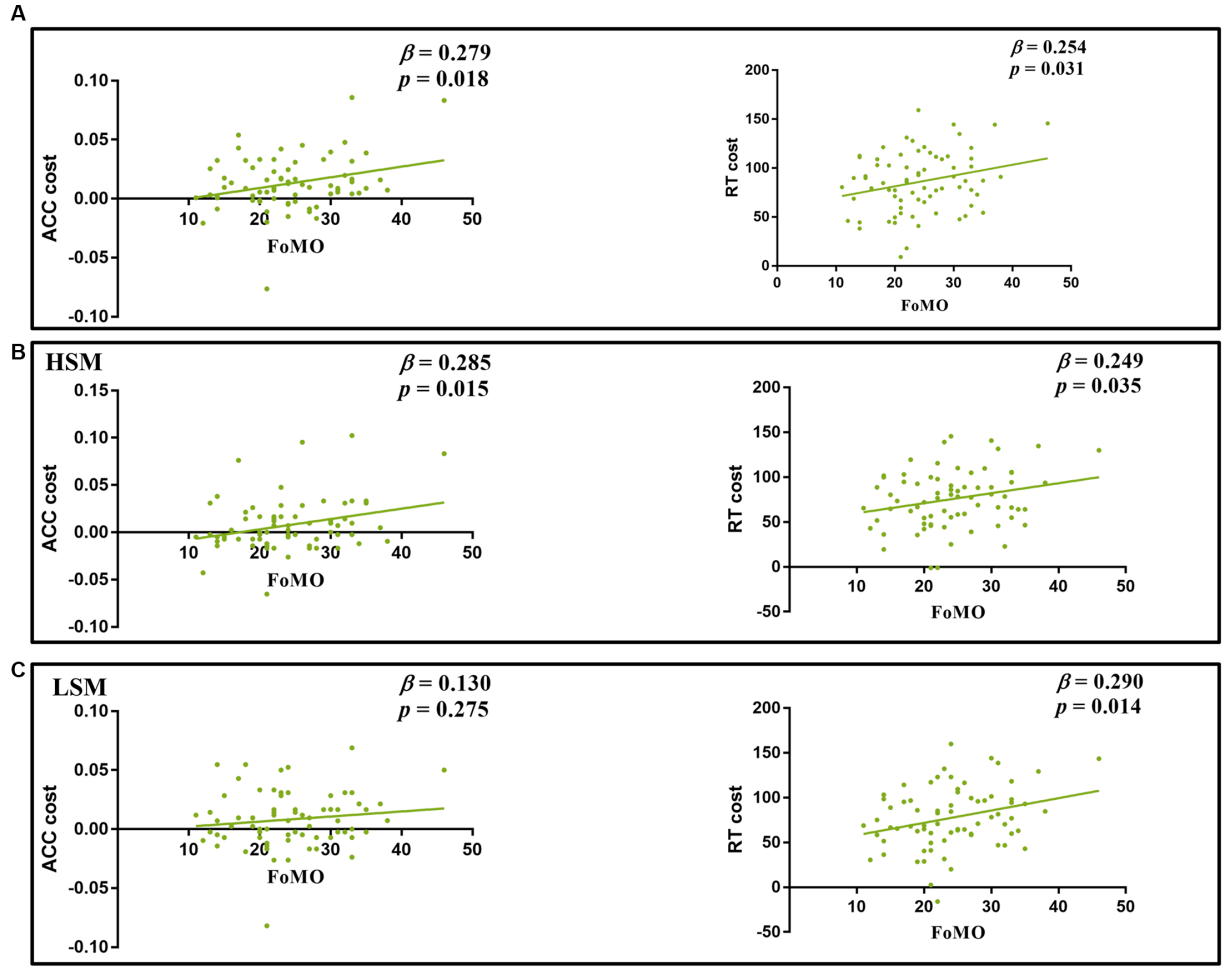
**(A)** Regression analyses between FoMO and ACC cost and RT cost; **(B,C)** regression analyses of FoMO with ACC cost and RT cost in HSM and LSM pictures.

Linear regression was used to examine whether FoMO scores could predict ACC cost and RT cost. The results showed that FoMO scores positively predicted ACC cost and RT cost (*β* = 0.28, *t* = 2.43, *p* < 0.05, *β* = 0.25, *t* = 2.20, *p* < 0.05) (see Figure 6A). For social media stimuli, the results showed that FoMO scores positively predicted ACC cost and RT cost for HSM (*β* = 0.29, *t* = 2.49, *p* < 0.05, *β* = 0.25, *t* = 2.15, *p* < 0.05). FoMO scores only positively predicted RT cost for LSM (*β* = 0.29, *t* = 2.53, *p* < 0.05) (see Figures 6B,C). For neutral pictures, FoMO scores only positively predicted ACC cost (*β* = 0.26, *t* = 2.21, *p* < 0.05).

### 6.2. ERP results

**Figure 7:**
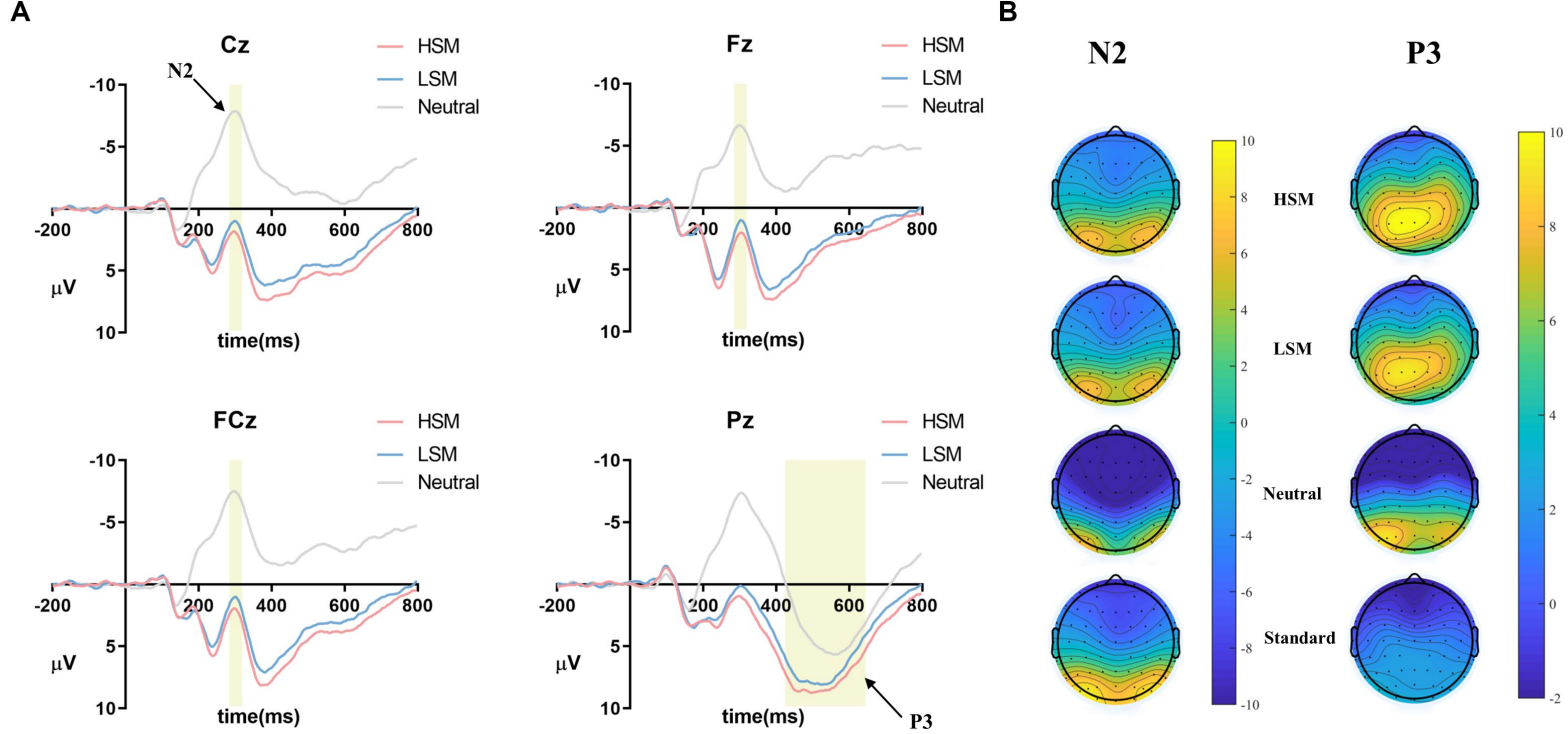
**(A)** ERPs in different picture types (deviant – standard) at electrode points (Fz, Cz, FCz, and Pz); **(B)** topographic maps for N2 (280–320 ms) and P3 (420–650 ms).

N2 and P3 amplitudes (deviant – standard) and topographic maps are displayed in Figures 7A,B.

#### 6.2.1. N2 component

**Figure 8:**
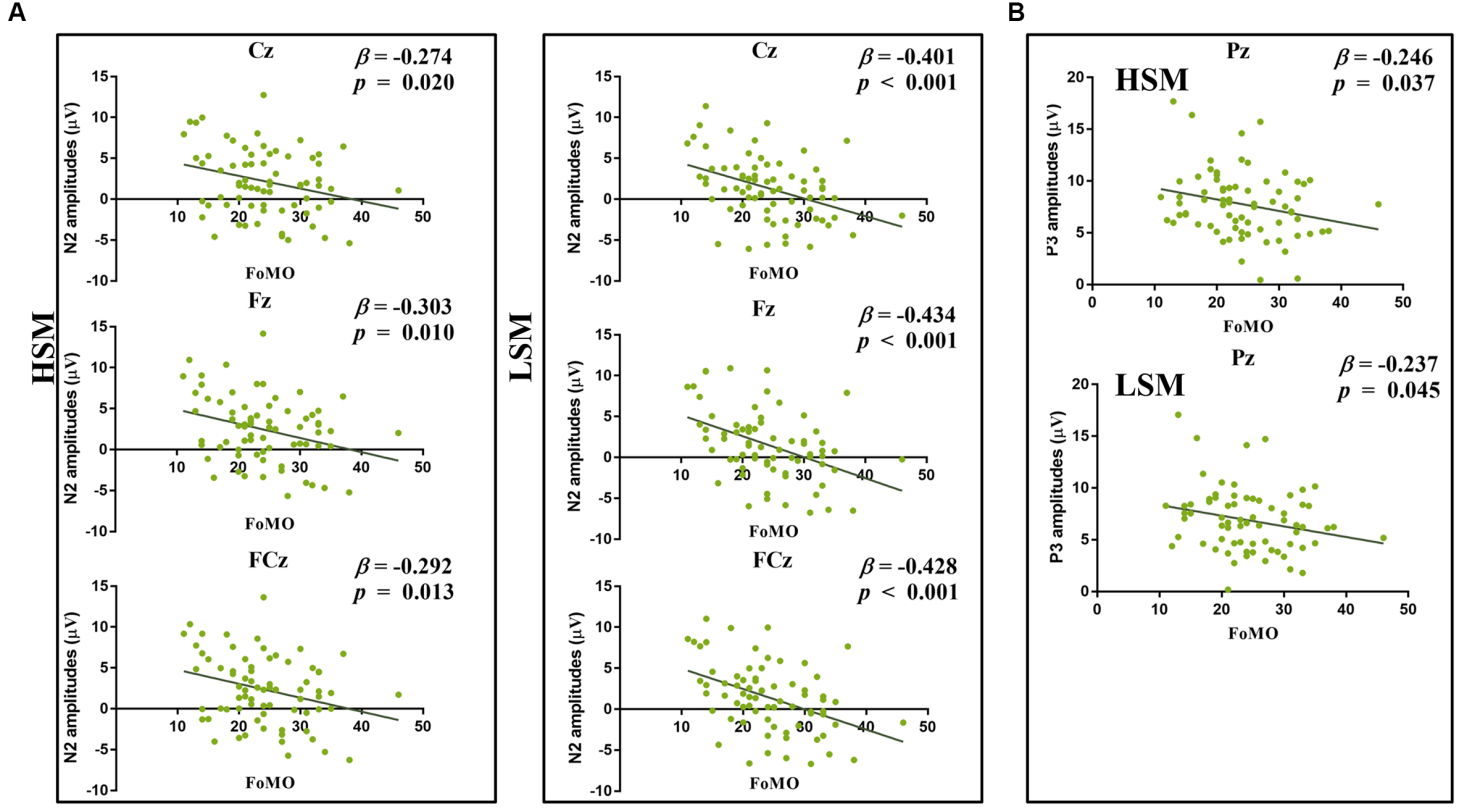
**(A)** Regression analyses between FoMO and N2 amplitudes (Cz, Fz, and FCz); **(B)** regression analyses between FoMO and P3 amplitudes (Pz).

The repeated-measures ANOVA revealed that the main effect of trial type was significant [*F* (2, 142) = 430.28, *p* < 0.001, *η*_p_^2^ = 0.86]. *Post-hoc* analyses revealed that N2 amplitude was larger for the neutral picture (*M* = −7.08 μV) than for the LSM picture (*M* = 1.44 μV) (*p* < 0.001) and larger for the LSM picture than the HSM picture (*M* = 2.33 μV) (*p* < 0.01). The main effect of electrode points was significant [*F* (2, 142) = 12.55, *p* < 0.001, *η*_p_^2^ = 0.15], and the trial type × electrode points was significant [*F* (4, 284) = 23.81, *p* < 0.001, *η*_p_^2^ = 0.25]. Furthermore, the linear regression was used to examine whether FoMO scores could predict the N2 amplitude (deviant – standard). The results showed that FoMO scores positively predicted N2 amplitude for HSM (Cz, Fz, and FCz) (*β* = −0.30, *t* = −2.66, *p* < 0.05; *β* = −0.27, *t* = −2.38, *p* < 0.05; *β* = −0.29, *t* = −2.53, *p* < 0.05, and LSM, *β* = −0.43, *t* = −4.03, *p* < 0.001; *β* = −0.40, *t* = −3.66, *p* < 0.001; *β* = −0.43, *t* = −3.96, *p* < 0.001) (see Figure 8A). FoMO scores did not significantly predict N2 amplitudes in neutral pictures.

#### 6.2.2. P3 component

The repeated-measures ANOVA revealed that the main effect of picture type was significant [*F* (2, 142) = 101.40, *p* < 0.001, *η*_p_^2^ = 0.59]. *Post-hoc* analyses revealed that P3 amplitude was larger for the LSM picture (*M* = 6.89 μV) than for the neutral picture (*M* = 4.02 μV) (*p* < 0.001) and larger for the HSM picture (*M* = 7.75 μV) than the LSM picture (*p* < 0.001). The linear regression was used to examine whether FoMO scores could predict the P3 amplitude (deviant – standard). The results showed that FoMO scores positively predicted P3 amplitude for HSM (Pz) (*β* = −0.25, *t* = −2.13, *p* < 0.05) and LSM (*β* = −0.24, *t* = −2.04, *p* < 0.05) (see Figure 8B), but FoMO scores did not significantly predict P3 amplitude in neutral pictures (*p* > 0.05).

## 7. Discussion

The current study revealed that FoMO undermines inhibitory control within the context of social media. As FoMO scores increased, participants showed larger N2 amplitude and smaller P3 amplitude when exposed to social media-related pictures. Individuals with FoMO direct limited cognitive resources toward relevant cues, resulting in decreased inhibitory control (33). These results were consistent with the findings that relevant cues are more likely to attract the attention of individuals and further generate poor inhibitory control (23).

## 8. General discussion

This study aimed to investigate how FoMO affects inhibitory control in the context of social media through two studies. The findings revealed that FoMO had no significant impact on general inhibitory control. However, within the context of social media, participants exhibited a decline in stimulus-specific inhibitory control as their FoMO scores increased, particularly when exposed to HSM pictures. Importantly, significant findings were observed at the electrophysiological level. When faced with different cues, participants showed smaller N2 amplitude and larger P3 amplitude under the cues of social media-related pictures compared to neutral pictures. Additionally, the N2 amplitude of social media-related pictures increased while the P3 amplitude decreased as the FoMO scores increased.

In Study 1, no significant effects of FoMO on general inhibitory control were observed at both the behavioral and electrophysiological levels. This result contradicts our first hypothesis. Nonetheless, in Study 2, when social media-related cues were introduced, we observed a significant and positive relationship between FoMO scores and both ACC cost and RT cost. This implies that as FoMO scores increase in the context of social media, inhibitory control diminishes, particularly in the HSM condition. This finding indicates that individuals with FoMO may not be notably affected by simple stimuli, such as the letters “W” and “M” used in Study 1. Previous research indicates that highly anxious individuals perform poorly on cognitively demanding tasks that require efficient cognitive processing (34, 35). Consequently, in Study 2, the inclusion of social media-related cues increased the task difficulty, and individuals with FoMO were required to allocate top-down attentional control resources to complete the task. This increased cognitive demand resulted in reduced inhibitory control.

On the electrophysiological level, the present study found that the N2 amplitude of social media pictures was smaller than that of neutral pictures, and the N2 amplitude of HSM pictures was smaller than that of LSM pictures. The N2 amplitude served as an indicator of cognitive resources expended during conflict detection (36). These findings suggest that individuals invest fewer cognitive resources in the early conflict detection stage when confronted with social media pictures. A previous study suggested that Internet addiction disorder students had lower cognitive resources in the conflict detection stage than the normal group (smaller N2 amplitude). This is because they had to engage in more cognitive endeavors to complete the inhibition task in the late stage (37). This is consistent with our findings. Furthermore, in this study, the P3 amplitude of social media pictures was larger than that of neutral pictures, and the P3 amplitude of HSM pictures was larger than that of LSM pictures. The increase in P3 amplitude indicated that more cognitive resources needed to be consumed to invest in the response process (28). This illustrates that when confronted with social media pictures, especially HSM pictures, they must allocate more cognitive resources to effectively inhibit the motor system in the later stages of inhibitory control.

Importantly, the results of the present study revealed a significant correlation between FoMO scores and the amplitudes of N2 and P3. As FoMO scores increased, the N2 amplitude increased and the P3 amplitude decreased, specifically for social media pictures but not for neutral pictures. This finding is consistent with the attentional control theory (5), which suggests that the inhibitory function of anxious individuals is particularly inefficient in the presence of distractors. High-anxious individuals are more negatively affected by distractors compared to low-anxious individuals (4). High-trait anxiety individuals showed a larger N2 amplitude than low-trait anxiety individuals, which means that high-trait anxious individuals use more cognitive resources to perform the monitoring process (38). The present study demonstrated that individuals with higher levels of FoMO consume more cognitive resources in the early conflict detection process of inhibitory control when confronted with social media-related cues (larger N2). However, as cognitive resources were limited (33), excessive consumption of resources during the early stage of conflict monitoring can lead to insufficient resources during the motor inhibition stage (smaller P3). Consequently, it leads to a decrease in the inhibitory control of the individual. When social media cues are added, individuals show a decrease in deficient stimuli-specific inhibitory control.

Several limitations require further attention. First, the study focused on measuring inhibitory control in young adults, specifically those around 20 years old. This may limit the generalizability of the findings to other age groups. A previous study found that FoMO did not appear in a particular age group (39). Thus, the generalizability of the results of the current study will have to be tested in future studies. Second, the use of self-report measures to assess FoMO may introduce potential biases and subjectivity. Future studies could incorporate more objective measures to complement self-reported data. Third, the present study found that the effect of FoMO on inhibition in the social media context and the adverse effects of anxiety on processing efficiency depended on inhibition and shifting (5). Shifting, as a component of executive functions, also plays a crucial role in cognitive processes. Thus, future research could further investigate the effects of FoMO on shifting in the social media context. Furthermore, there are some potential issues in the material selection in Study 2, such as the limited distinctions between high and low social media stimuli. Future studies could perhaps replace low social media stimuli with stimuli unrelated to social media to increase the differentiation among the three kinds of pictures.

## 9. Conclusion

The present study has revealed that FoMO had an effect on stimulus-specific inhibitory control. Specifically, FoMO undermines inhibitory control in the social media context. As FoMO scores increased, individuals’ inhibitory control abilities were found to decrease when exposed to social media-related cues. FoMO exerts an impact on inhibitory control by consuming more cognitive resources during the early conflict detection stage, while the later stages of the inhibitory process suffer from insufficient cognitive resources. These findings provide insights into the underlying mechanisms through which FoMO influences inhibitory control processes in the social media context.

## Data availability statement

The raw data supporting the conclusions of this article will be made available by the authors, without undue reservation.

## Ethics statement

The studies involving humans were approved by Institute of Brain and Psychological Sciences, Sichuan Normal University. The studies were conducted in accordance with the local legislation and institutional requirements. The participants provided their written informed consent to participate in this study.

## Author contributions

YX: Formal analysis, Investigation, Visualization, Writing – original draft, Writing – review & editing. YT: Conceptualization, Funding acquisition, Writing – review & editing.
